# Culture, meat, and cultured meat

**DOI:** 10.1093/jas/skaa172

**Published:** 2020-08-03

**Authors:** Christopher J Bryant

**Affiliations:** Department of Psychology, University of Bath, Claverton Down, Bath, UK

**Keywords:** cultured meat, food technology, meat alternatives, regulation, religion, social institutions

## Abstract

Cultured meat grown in vitro from animal cells has the potential to address many of the ethical, environmental, and public health issues associated with conventional meat production. However, as well as overcoming technical challenges to producing cultured meat, producers and advocates of the technology must consider a range of social issues, including consumer appeal and acceptance, media coverage, religious status, regulation, and potential economic impacts. Whilst much has been written on the prospects for consumer appeal and acceptance of cultured meat, less consideration has been given to the other aspects of the social world that will interact with this new technology. Here, each of these issues is considered in turn, forming a view of cultured meat as a technology with a diverse set of societal considerations and far-reaching social implications. It is argued that the potential gains from a transition to cultured meat are vast, but that cultural phenomena and institutions must be navigated carefully for this nascent industry to meet its potential.

## Introduction

Our current meat production system is resource-intensive, has negative environmental impacts, entails animal suffering, and is linked to a number of public health issues, including animal-transmitted pandemics and antibiotic resistance (Mathew et al., 2007; Oliver et al., 2011; Lymbery and Oakshotte, 2014; IPCC, 2018). Yet, global demand for meat is forecast to increase rapidly as the world population grows (McLeod, 2011).

One proposed solution to decrease our consumption of meat from animals is the development and utilization of cultured meat, which can be grown from animal cells without animal slaughter (Post, 2012). In addition to eliminating the need for animal slaughter, cultured meat is associated with far less harm to the environment in terms of greenhouse gas emissions and land and water use (Tuomisto, 2019). Cultured meat could become available commercially within a few years (Lucas, 2019).

Recent years have seen a proliferation of research on consumer acceptance of cultured meat (Bryant and Barnett, 2018, 2019; van der Weele and Driessen, 2019; Wilks et al., 2019). However, Stephens et al. (2018) have argued that the social discourse around cultured meat must move beyond narrow conceptions of consumer acceptance and consider broader societal issues. Therefore, this article will consider a range of important cultural phenomena and institutions that will interact with cultured meat: media coverage, religions, regulations, and economic impacts.

## Media Coverage

The media is an important source of information to the public and likely plays a crucial role in shaping public perceptions of food technologies (Frewer et al., 1995). Indeed, there is some evidence that media coverage of cultured meat shapes public opinion by highlighting certain aspects of the concept (Laestadius and Caldwell, 2015).

Much of the early media coverage of cultured meat in the United States and Europe has been neutral or positive, frequently discussing the challenges with conventional animal agriculture and the relative benefits of cultured meat in terms of animal welfare, the environment, food security, and human health (Goodwin and Shoulders, 2013). This positive coverage is likely due, in part, to the sources of information used; these included New Harvest, People for the Ethical Treatment of Animals, cultured meat researchers, and academics (Goodwin and Shoulders, 2013).

This may partially explain the more positive attitudes of those who are more familiar with the concept because they presumably become familiar through the media. Bekker et al. (2017) found that positive or negative information about cultured meat shifted individuals’ opinions in that direction, but that those who were already familiar with the concept were less influenced by the information. Therefore, this early positive coverage is certainly a good thing for cultured meat, because resulting positive attitudes will be likely to endure.

However, there are certainly aspects of the technology that invite unflattering media coverage. In an analysis of Australian print media, Dilworth and McGregor (2015) found that unnaturalness was the most commonly discussed theme. The authors speculate that stakeholders such as farming lobbies could capitalize on this narrative to undermine consumer acceptance, commenting that “embodied responses based on deeply entrenched ideas of food and nature are not easily overcome” (p. 103). Further, the authors identify a number of less common narratives that generally frame cultured meat in a negative way: that increasing reliance on technological solutions undermines the process of genuine social change, that meat production is a way of connecting with nature, and that continuing to instrumentalize animals in a way that simply sidesteps the issues may undermine the animal liberation movement. Hopkins and Dacey (2008) have discussed each of these arguments and concluded that they are not valid reasons to oppose cultured meat.

Interestingly, Hopkins (2015) demonstrated that media coverage of cultured meat has given undue focus to vegetarians’ opinions of cultured meat. This is despite their lower propensity to want to eat it compared with meat-eaters (Wilks and Phillips, 2017; Bryant et al., 2019), although they are more likely to recognize its benefits for animals and the environment (Wilks and Phillips, 2017). One could argue that, with the aim of reducing animal product consumption, it is unimportant whether vegetarians would eat cultured meat—indeed, the idea that cultured meat is “for vegetarians” could undermine its appeal to meat-eaters.

Bryant and Dillard (2019) demonstrate how different frames one might encounter in media coverage of cultured meat can affect consumer perceptions. The researchers found that those who saw a frame that emphasized the “high tech” elements of cultured meat were significantly less likely to want to eat it compared with those who saw frames that emphasized the societal benefits of cultured meat, or its sensory similarity to conventional meat. At this stage, it is likely that most of the coverage of cultured meat will frame it primarily as scientific discovery. This is because news on the topic is likely to relate to new advancements in the technology, and media coverage of conceptually similar food technologies has been primarily event-driven (Marks et al., 2003; Botelho and Kurtz, 2008).

## Religion

The religious status of cultured meat is an issue that has received attention in various religious communities and has been one strand of the wider cultural debate (Hopkins, 2015). Notably, this is an issue for the world’s 1.8 billion Muslims, 1.1 billion Hindus, half a billion Buddhists, and over 10 million Jews (Hackett and McClendon, 2017). Comprising almost half of the global population, these people all follow religions with specific rules and customs around meat consumption.

Survey data from nationally representative samples of 3,030 people in the United States, India, and China (Bryant et al., 2019) contain data from Jews (*n* = 23), Muslims (*n* = 193), Hindus (*n* = 730), and Buddhists (*n* = 139) on which cultured meat products they would be willing to eat. This can give some empirical insight into the views of adherents to these various religions. Respondents in this study were given the following description of “clean meat”:

*One food innovation is called clean meat. This type of meat is identical at the cellular level to conventional meat. This is real meat grown directly from animal cells. Clean meat is produced in a clean facility, similar to a brewery. The process does not involve raising and slaughtering farm animals. The final product has an identical taste and texture to conventional meat. Clean meat offers significant benefits for human health, the environment, and animal welfare. Several companies have already successfully produced and taste-tested clean meat. The products will be available for retail purchase in 1 to 5 yr.*

### Judaism

In Judaism, most rabbis agree that cultured meat itself is kosher, though some say the cells must come from a kosher-slaughtered animal (Bleich, 2013; Kenigsberg and Zivotofsky, 2020). Indeed, the rabbi who will ultimately decide whether cultured meat is kosher via the Orthodox Union’s kosher certification scheme, the largest in the world (Fischer, 2016), appears enthusiastic about the concept (Purdy, 2018). However, there are interesting questions about whether cultured meat could allow kosher-observing Jews to consume otherwise prohibited foods.

The first question is whether cultured meat consumed with dairy would be kosher. The second is whether cultured pork would be kosher. Both rest on the question of whether cultured meat is considered to be meat in a religious sense, or is pareve, meaning it is considered to be something other than meat or dairy (Sokol, 2013; McDonald, 2018). For a more complete discussion of the kosher status of cultured meat, see Kenigsberg and Zivotofsky (2020). 

**Table 1.** Percentage of Jews who eat/would eat each species of meat (data from Bryant et al., 2019)

Judaism (*n* = 23)			
	Currently eat, %	Find cultured meat appealing, %	Difference, %
Beef	87.0	69.6	−17.4
Poultry	91.3	69.6	−21.7
Pork	60.9	60.9	0
Lamb/Goat	65.2	60.9	−4.3

Amongst the 23 Jewish people in Bryant et al.’s (2019) survey data, 61% said they currently ate pork, and 61% said they would eat cultured pork. This was slightly lower than the proportion who would eat cultured beef (70%) or chicken (70%), but still a majority. Notably, pork was the only meat for which there was no overall preference for conventional meat—for all other species, fewer respondents said they would eat cultured meat than currently ate conventional meat. Our data did not allow us to interpret whether participants would eat cultured meat and dairy together.

### Islam

In Islam, the relevant question is whether cultured meat is halal. Hamdan et al. (2018) argue that, based on Quran scripture and interpretation by prominent Islamic jurists, cultured meat is halal if the cells used are from a halal-slaughtered animal and no blood or animal-based serum is used in the production process. However, since the origin of the cells is central to the halal status of cultured meat, halal meat from pigs and other haram species is unlikely to be approved (Purdy, 2018).

Indeed, survey data appear to confirm this: of 193 Muslims, 58% would eat cultured beef, 68% would eat cultured lamb or goat meat, and 49% would eat cultured chicken, but only 28% would eat cultured pork (Bryant et al., 2019).

**Table 2.** Percentage of Muslims who eat/would eat each species of meat (data from Bryant et al., 2019)

Islam (*n* = 193)			
	Currently eat, %	Find cultured meat appealing, %	Difference, %
Beef	64.8	57.5	−7.3
Poultry	74.6	48.7	−25.9
Pork	30.1	27.5	−2.6
Lamb/Goat	81.3	67.9	−13.4

As in Judaism, a significant proportion of adherents to Islam indicated that they do eat conventional pork, despite this being prohibited in the religion. This highlights the fact that many people of all different religions do not strictly follow the prescribed dietary guidelines (Rarick et al., 2011).

### Hinduism

Many Hindus interpret *ahimsā*, the principle of nonviolence, as requiring vegetarianism, although this is not explicit in Hindu texts (Dudek, 2013). The focus on nonviolence means that vegetarian Hindus are likely to see cultured meat as a way of avoiding harming animals, and some may decide it is permissible to eat. Some have suggested that cultured beef is unlikely to be accepted in Hinduism because cows are considered sacred (Mattick et al., 2015).

**Table 3.** Percentage of Hindus who eat/would eat each species of meat (data from Bryant et al., 2019)

Hinduism (*n* = 730)			
	Currently eat, %	Find cultured meat appealing, %	Difference, %
Beef	18.2	18.9	+0.7
Poultry	67.5	68.1	+0.6
Pork	18.5	19.6	+1.1
Lamb/Goat	61.4	64.4	+3.0

Again, survey data appear to confirm this. Of 730 Hindus in the dataset, 65% would eat cultured goat and 68% would eat cultured chicken, but only 20% would eat cultured pork and 19% would eat cultured beef (Bryant et al., 2019). Interestingly, Hindus were the only religious group who were overall more willing to eat cultured meat than conventional meat for all relevant species, perhaps highlighting the motivation to avoid harming animals. Notably, just 24% of the Hindus in this dataset were vegetarian, again marking a departure from the diets we might expect in this religious group.

### Buddhism

Less has been written about the permissibility of cultured meat in Buddhism. Though many practicing Buddhist monks refrain from eating meat, only 1.4% of those identifying as Buddhist (most of whom were in China) were vegetarian or vegan in this data (Bryant et al., 2019). That said, 81% would eat cultured beef, 73% would eat cultured pork, 66% would eat cultured goat, and 61% would eat cultured chicken.

**Table 4.** Percentage of Buddhists who eat/would eat each species of meat (data from Bryant et al., 2019)

Buddhism (*n* = 139)			
	Currently eat, %	Find cultured meat appealing, %	Difference, %
Beef	87.8	81.3	−6.5
Poultry	82.0	61.2	−20.8
Pork	81.3	73.4	−7.9
Lamb/Goat	69.8	65.5	−4.5

Overall, we observe a majority of religious consumers being open to eating cultured meat in principle, with some evidence of avoidance of cultured meat from species that are not allowed in the religion (e.g., pork in Islam and beef in Hinduism). That said, a sizable portion of respondents in all religions appeared not to adhere strictly to the diets prescribed by their religion, meaning that many nominally religious people are unlikely to be sensitive to religious rulings on the permissibility of cultured meat per se.

## Regulation

Recent years have seen increasing clarity over the regulatory frameworks for marketing cultured meat in the European Union and the United States. However, some important issues are yet to be addressed. A central issue in both markets is whether cultured meat will be considered meat.

### European Union

In Europe, cultured meat will likely require approval from the European Food Safety Authority (**EFSA**) under the Novel Foods Regulation (EU) No (2015/2283) (Merten-Lentz, 2018; Froggart and Wellesley, 2019; Verzijden, 2019). This regulation is primarily designed to ensure that new foods are safe to consume, labeled properly so as not to mislead consumers, and not nutritionally disadvantageous compared with existing food they seek to replace (European Commission, n.d.). It is not yet clear what type of nutritional and toxicological evidence EFSA would require to approve cultured meat. Moreover, since there is no pre-market consultation process, it is likely that producers in Europe will have to “learn by doing” through EFSA applications (Verzijden, 2019).

As Froggart and Wellesley (2019) have argued, it is likely that cultured meat products will be required to carry a name or label that clearly specifies the production process. The Food Information to Consumers Regulation (2011/1169) requires that food labeling is clear, precise, and easily understandable (European Commission, 2016). Newly approved novel foods, meanwhile, may be subject to further labeling requirements under the Novel Foods Regulation (Froggart and Wellesley, 2019).

Additionally, there are some questions about whether cultured meat will be able to be marketed as meat (Froggart and Wellesley, 2019). The Food Information to Consumers Regulation currently defines meat as “skeletal muscles of mammalian and bird species recognized as fit for human consumption with naturally included or adherent tissues.” Skeletal muscle, in turn, is defined as “muscles under the voluntary control of the somatic nervous system.” (European Commission, 2016). The current definition, therefore, would seem to exclude cultured meat.

If cultured meat products contain ingredients that are genetically modified, they will instead be subject to Regulation (EC) No 1829/2003 on genetically modified food and feed (Froggart and Wellesley, 2019). Decisions taken under this regulation are based on risk assessments as well as public acceptance and economic considerations. In practice, cultured meat products with a genetically modified component are less likely to be permitted in Europe, given heavy restrictions on genetically modified foods already in place and generally poor public perceptions of the technology (Eurobarometer, 2010).

Whilst cultured meat would be approved at the level of the European Union, it is likely that any required inspections and enforcement would be carried out by member states (Verzijden, 2019). European producers, therefore, need to be aware of both European and national legislation.

### United States

There is somewhat less clarity around the regulatory framework in the United States. Verzijden (2019) has identified some of the major differences from the EU as being the presence of a pre-market consultation mechanism, consistency in the bodies regulating and enforcing the regulation, and the shared jurisdiction of cultured meat regulation between the U.S. Department of Agriculture (**USDA**) and the Food and Drug Administration (**FDA**). At present, it seems that the FDA will regulate the pre-harvest production process and materials, and the USDA will regulate post-harvest processes including monitoring and labeling. However, as Verzijden (2019) points out, the existing agreement between these bodies is not binding, and the situation may, therefore, change. Moreover, individual states may have additional regulations.

Cultured meat may fall outside of the Federal Meat Inspection Act’s definition of meat, which defines meat as coming from an animal carcass (Sanchez, 2018). While this was thought to be determinative of which agency should have jurisdiction over cultured meat regulation, it appears that this is no longer the case. However, this is still a relevant issue for the question of whether cultured meat will be allowed to be marketed as meat.

In both the United States and the EU, cultured meat may not be defined as “meat” under existing regulations. However, it is possible that such definitions will be revised to include cultured meat, especially given the presence of health and allergy concerns (Simon, 2018). Although meat allergy is rare in adults, a significant portion of potential consumers could have allergic reactions to eating meat, especially beef and poultry (Restani et al., 2009; one particularly common form of red meat allergy is an alpha-gal syndrome. This is caused by an immune system reaction to a sugar molecule that can enter the blood through tick bites (Mayo Clinic, 2019). It may be possible for cultured meat to be engineered to exclude alpha-gal, thus making products appropriate for alpha-gal syndrome sufferers, but further research on this is needed) Since cultured meat is meat on a molecular level, it is extremely likely that those who are allergic to certain types of meat will also be allergic to cultured meat. Labeling that fails to adequately describe a product could lead to serious health risks for consumers (Watson, 2018). However, there are increasing attempts from meat industry incumbents to prohibit the term “meat” from being used in the labeling of cultured meat products (Flynn, 2019).

## Economic Impacts

One area worthy of further discussion is the potential economic impacts that cultured meat will have. There are concerns around the impact of cultured meat on animal farmers, the potential for the consolidation of food production under large corporations, and concerns about how the relative price of cultured meat could impact inequality (Bonny et al., 2015; Stephens et al., 2018).

### Agricultural employment

Concerns about the impact of cultured meat on animal farmers are evident in various legislative attempts to restrict cultured meat (Flynn, 2019). Indeed, cultured meat and related technologies may eventually replace livestock farming (Phillips and Wilks, 2019). Whilst just 4.4% of EU employment is in agriculture (Eurostat, 2017), this percentage is much higher in less developed parts of the world (Roser, 2019). Moreover, many of those who work in agriculture are concentrated in rural areas where the economy is largely dependent on agriculture (Kurrer and Lawrie, 2018).

Whilst cultured meat production will no doubt create new jobs, these would require an entirely different set of skills to current agricultural workers, who tend to have a lower level of education than the general population (Eurostat, 2017). Bonny et al. (2015) have argued that animal farmers may end up providing a small and premium niche of the overall meat market. They may respond by adopting agroecology concepts to improve sustainability and/or adopting biotechnologies such as cloning and genetic modification. Alternatively, they may switch to producing crops for human consumption or biofuels (Kurrer and Lawrie, 2018).

Nonetheless, it is likely that a significant shift toward cultured meat production and away from conventional animal agriculture will mean that many people currently employed in animal agriculture lose their jobs. This is, of course, a problem for these individuals. However, it is self-evidently untenable in the long run to insist that all of the existing jobs in any given sector must continue to exist.

There are countless examples throughout the history of jobs that technology rendered obsolete. Most notably, the Luddites of the textile industry in England in the 19th century destroyed machinery to protest against the job losses the technology created. Likewise, a “knocker-upper” was someone who was employed to knock on workers’ windows to wake them up before alarm clocks were widespread. There is no doubt that the individuals in these occupations would have been worse off without those jobs—but does anybody today think that society would be better off if textile machinery or alarm clocks had been banned to save them? One could, of course, create many more jobs in farming tomorrow by banning combine harvesters. Deliberately pursuing less efficient production in order to create or preserve jobs in a free market is neither sustainable nor desirable.

This point is perhaps best illustrated by an allegory about an economist visiting a country with a planned economy. On visiting a construction site, the economist noticed that the project had employed hundreds of workers with shovels instead of using any modern machinery or equipment. He asked why there were no machines, and the foreman told him that this way, more jobs were created. The economist responded that if the objective was to create jobs rather than finish the construction project, they should take away the workers’ shovels and have many more workers with teaspoons instead (Tanner, 2015).

### Consolidating food production

Others have expressed concerns about the consolidation of food production under a smaller number of actors with greater capital (Driessen and Korthals, 2012; Hocquette, 2016). Indeed, consolidation in the food industry, in general, could result in oligopolies exerting pressure on suppliers, limiting consumer choice, and driving industrialization (Heinrich Böll Stiftung, 2017). Perhaps more pertinently still, cultured meat production may only be feasible in countries with sturdy energy infrastructure and a highly educated workforce. This has led Hocquette (2016) and Stephens et al. (2018) to speculate that cultured meat could exacerbate economic inequality between countries, as well as within.

However, it is not yet clear what shape the cultured meat industry will take. As with many of the social questions, this is dependent on as-yet-unknown aspects of the technology: for instance, van der Weele and Driessen (2013) offer the alternative “pig-in-the-backyard” vision where the technology is democratized and communities can produce their own meat from locally kept animals. In any case, the production of cultured meat will require the production of inputs for culture media, and it is possible that these inputs could still be produced in existing agricultural systems.

### Consumer inequality

Finally, some have worried that cultured meat may exacerbate inequalities between the rich and the poor (Cole and Morgan, 2013; Bonny et al., 2015; Stephens et al., 2018). Bonny et al. (2015) have speculated that cultured meat could feed the masses cheaply, leaving real meat the preserve of the wealthy. Conversely, Cole and Morgan (2013) have worried that cultured meat, being substantially more expensive than conventional meat, would allow the wealthy to eat meat without moral consequence, leaving only the poor reduced to killing animals for their food.

Interestingly, the economics of cultured meat production means that both of these visions may hold some truth. Whilst the cost of producing cultured meat has fallen rapidly in recent years, it is likely that it will still be more expensive than its conventional counterpart when it first comes to market (González and Koltrowitz, 2019). Some commentators believe it will first be available at a premium price in restaurants only (Purdy, 2019). During this stage, cultured meat may be seen as a luxury or novelty only available to the rich or those with access to exclusive outlets. Given these conditions, consuming cultured meat could convey wealth and status.

However, in the longer term, cultured meat will become cheaper to produce and could be cheaper than conventional meat if it is made more efficiently (Fountain, 2013). At this time, any prestige associated with cultured meat consumption will likely be diminished as it becomes commonplace. Indeed, we have seen the same process play out with other foods: salt, now ubiquitous and even maligned, was once so valuable that it was used to pay soldiers (Salt Association, n.d.).

The cost of producing cultured meat is likely to be relatively high initially but decreases over time. This will likely mean that it is, at first, only available to affluent consumers, but may become increasingly common as the price falls. The price of cultured meat production falling below the price of conventional meat production may represent a tipping point for meat production worldwide.

## Conclusion

Cultured meat is a technology with the potential to alleviate the ethical, environmental, and public health concerns associated with conventional meat production, including greenhouse gas emissions, land and water use, antibiotic resistance, food-borne and zoonotic diseases, and animal slaughter. However, beyond overcoming technical challenges in perfecting and scaling up the production process, producers and advocates of cultured meat must consider their relation to a range of social and cultural phenomena and institutions. 

These two sets of challenges are inextricably linked because many of the uncertainties around regulation, religious classification, and economic impacts relate to specific elements of the production process that are unknown or proprietary. For example, the use of animal serum has implications for the halal status of cultured meat, while the scalability of production processes has implications for the shape of the cultured meat industry.

With respect to regulation and religious dietary restrictions, one can easily lose sight of the original objectives. For example, the European Union’s Novel Food Regulation has ensuring food safety as a central objective, yet related legislation might mean cultured meat cannot be labeled as meat, putting consumers with allergies at risk. Similarly, the halal slaughter was originally conceived based on the principle of reducing animal suffering (Withnall, 2014), yet, it may now require animals used in cultured meat production to be killed for their meat to be permissible in Islam (Hamdan et al., 2018). One must hope that the spirit of the law will prevail over the letter of the law in these cases.

Cultured meat producers face a range of technical challenges, and many of these are upstream from social challenges. It is important to consider how cultured meat might interact with these important cultural forces, and in some cases production decisions can be made to optimize outcomes with regard to the societal issues discussed here. Careful navigation of these challenges can ensure that cultured meat can fulfill its potential to alleviate animal suffering and environmental degradation.

## Abbreviations

EFSA: European Food Safety Authority
FDA: Food and Drug Administration
USDA: U.S. Department of Agriculture
